# Allogeneic hematopoietic stem cell transplantation in the COVID-19 era

**DOI:** 10.3389/fimmu.2023.1100468

**Published:** 2023-02-22

**Authors:** Jonathan Bordat, Sébastien Maury, Mathieu Leclerc

**Affiliations:** ^1^ Hematology Department, Henri Mondor Hospital, Assistance Publique/Hôpitaux de Paris, Créteil, France; ^2^ Institut Mondor de Recherche Biomédicale, équipe Immunorégulation et Biothérapies, INSERM U955, Créteil, France; ^3^ Faculté de Médecine, Paris-Est Créteil University, Créteil, France

**Keywords:** COVID-19, hematopoietic stem cell transplantation, vaccination, SARS-CoV-2, immune response

## Abstract

Allogeneic hematopoietic stem-cell transplantation (allo-HSCT) recipients are especially vulnerable to coronavirus disease 19 (COVID-19), because of their profound immunodeficiency. Indeed, the first pandemic wave was marked by a high mortality rate in this population. Factors increasing immunodepression such as older age, immunosuppressive treatments or a short delay between transplant and infection appear to worsen the prognosis. Many changes in clinical practice had to be implemented in order to limit this risk, including postponing of transplant for non-malignant diseases, preference for local rather than international donations and for peripheral blood as stem cell source, and the widespread use of cryopreservation. The great revolution in the COVID-19 pandemic came from the development of mRNA vaccines that have shown to be able to prevent severe forms of the disease. More than 75% of allo-HSCT recipients develop seroconversion after 2 doses of vaccine. Multiple studies have identified lymphopenia, exposure to immunosuppressive or anti-CD20 therapies, and a short post-transplant period as factors associated with a poor response to vaccination. The use of repeated injections of the vaccine, including a third dose, not only improves the seroconversion rate but also intensifies the immune response, both in B cells and T cells. Vaccines are an effective and well-tolerated method in this high-risk population. Some studies investigated the possibility of immune protection being transferred from a vaccinated donor to a recipient, with encouraging initial results. However, dynamic mutations and immune escape of the virus can lead to breakthrough infections with new variants in vaccinated individuals and still represent a threat of severe disease in allo-HSCT recipients. New challenges include the need to adapt vaccine protection to emerging variants.

## 1 Introduction

Coronavirus disease 19 (COVID-19), caused by severe acute respiratory syndrome coronavirus 2 (SARS-CoV-2), appeared in 2019, rapidly spread all over the world and was declared a pandemic disease by WHO in March 2020. This fatal disease, which cumulates currently more than 660 million cases and more than 6.7 million deaths (on January, 16^th^ 2023, according to the WHO declaration (1)), had an important impact on hospital organization and protection of vulnerable patients. Allogeneic hematopoietic stem-cell transplantation (allo-HSCT) recipients are especially vulnerable to infectious diseases, because of their profound immunodeficiency induced by a nascent immune system exposed to immunosuppressive drugs. In this high-risk population, the need for protection from this pandemic has led to substantial modifications in daily clinical practice.

In this review, we are discussing the impact of COVID-19 on the management of allo-HSCT recipients and focusing on the determinants and specificities of immune response to SARS-CoV-2 infection and/or vaccination in this high-risk population.

## 2 Clinical outcome in allo-HSCT patients with COVID-19

### 2.1 Clinical characteristics

In studies focused on allo-HSCT recipients (2–6), most of which were conducted during the first wave of the pandemic, the most frequent symptoms at diagnosis of SARS-CoV-2 infection were fever (65-75%), cough (55-65%), upper respiratory symptoms (20 – 45%) and asthenia (10-49%). Some patients also developed flu-like syndrome, myalgia, digestive disorders or neurological symptoms (anosmia, dysgeusia…). Despite most of patients were symptomatic, above 10% had no symptom at diagnosis of COVID-19. More than one-third (32-52%) required supplemental oxygen therapy (2, 4, 7). In the large European Society for Blood and Marrow Transplantation (EBMT) study (2) with 382 patients, no differences in clinical symptoms were reported between allo-HSCT and autologous transplant recipients with the same clinical symptoms at diagnosis (2). Piñana et al. (5), compared the clinical presentation of non-transplant patients with hematologic malignancies (60% lymphoid malignancies) and allo-HSCT recipients diagnosed positive for COVID-19 from March 1, 2020, to May 15, 2020 and no significant differences were found, with the same number of patients symptomatic above 90% in each group, presenting similar symptoms, mainly fever, asthenia and cough. These similar results may be due to a common immunosuppressed status in these populations.

Resolution of COVID-19 symptoms in allo-HSCT recipients takes a median time ranging from 14 to 26 days (2, 5, 7). Despite resolution of clinical symptoms, 5.6% (13/231) of patients in the EBMT study by Ljungman et al. (2) remained PCR positive, highlighting the problem of long excretory patients in this immunocompromised population. Another problem reported in the European Conference on Infections in Leukaemia (ECIL)-9 guideline (8) is the long COVID-19 syndrome (or post-acute COVID-19 syndrome) defined by the persistence of delayed or long-term symptoms and/or complications beyond 4 weeks after the start of the acute phase. This syndrome presents with persistent symptoms associated with COVID-19, including fatigue, dyspnea, cough, chest pain, and impaired quality of life. Few studies have investigated this disorder in patients with hematological malignancies and there is no specific data regarding its prevalence among allo-HSCT recipients. No treatment is currently approved for long excretory patients or long COVID-19 syndrome, leading to an impaired quality of life in this population.

### 2.2 An evolving high-risk disease

Initial studies reported a poor outcome of COVID-19 in patients with oncological or hematological diseases (5, 9, 10) particularly in allo-HSCT recipients, as summarized in **Table 1** (2–4, 7, 11–14), with a case fatality rate ranging from 22 to 32% (2, 4, 7). The multicenter study from EBMT and Grupo Español de Trasplante Hematopoyético y Terapia Celular (GETH) (2), had reported an observation of 236 allo-HSCT recipients and 146 autologous recipients infected with SARS-CoV-2 during the first pandemic wave, and found a mortality rate of 22% at 6 weeks in allo-HSCT recipients, with no difference in mortality between allogeneic and autologous recipients. In the pooled population, 74,4% were hospitalized with one third requiring oxygen therapy, and 22,5% requiring an intensive care unit (ICU) admission with a mortality rate as high as 55% at 6 weeks for these patients.

**Table 1:** Studies about COVID-19 infections in stem cell transplanted patients.

Author	Year of recruitement	N (allo / auto / carT)	Age (years)	Median time since transplant (months)	Hosp / ICUs / MV	OS	Mortality	Risk factors of mortality
LJUNGMAN et al. Leukemia 2021	2020	236 (62%) / 146 (38%) / 0	56.8	117.9 (range:0.9 - 350.3)	74,4% / 22,5% / NA	71,6% at 10 weeks	28,4% at 10 weeks	Multivariate analysis (mortality): *Age (HR 1.21; 95% CI: 1.03–1.43) *Higher ISI group° (HR 1.84; 95%CI: 1.02–3.33) *ICU admission (HR: 3.17; 95% CI: 2.00–5.01) *Protector: better performance status (HR 0.83; 95%CI: 0.74–0.93).
El FAKIH et al. Bone Marrow Transplantation 2021	2020	52 (57%) / 39 (43%) / 0	35	14.9 (IQR: 16.3–38.9)	53% / 14% / 10%	NA	4% (intubation 10%)	Multivariate analysis: *Time from HSCT to COVID-19 < 6 months (fdr severity)
VARMA et al. Leukemia 2020	2020	20 (59%) / 14 (41%) / 0	57	17,4	74% / 32% / 24%	NA	21%	Univariate analysis: *Older age *Being on steroids at diagnosis of COVID-19 <1 year of HSCT for allograft
CAMARGO et al. Transpl Infect Dis. 2021	2020	15 (53%) / 12 (43%) / 1(4%)	57	21,56 (IQR: 1,1- 41,9)	57,1% / NA / 25%	86% at 30d	14% at 30 days	Univariate analysis: *Older age *Timing from HSCT <12m *Intensity of immunosuppression
ALTUNTAS et al. Bone Marrow Transplantation 2021	2020	12 (37,5%) / 20 (62,5%) / 0	NA	NA	100% / 21,9% / 15,6%	NA	15.60%	NA
AGRAWAL et al. Indian J Hematol Blood Transfus 2022	2020	10 (36%) / 18 (64%) / 0	50.5	8,5 (range: 0,4-67,1)	53% / NA / 25%	85,7% at 28d	14.3% at 28 days	Univariate analysis: *<1 year of HSCT *Severe infection
Allo-SCT patients only								
Author	Year of recruitement	N (allo)	Age (years)	Median time since the graft in month	02 / ICUs / MV	OS	Mortality	Risks factors of mortality/severity
LJUNGMAN et al. Leukemia 2021	2020	236	54.1	15,8.	32,2% / NA / NA	77,9% at 6 weeks	22% at 6 weeks	Multivariate analysis (mortality) *Age at COVID-19 (HR:1.29; 95%CI: 1.05–1.58) *ICU admission (HR:4.42; 95%CI: 2.25–8.65) Multivariate analysis (severity) *Ongoing immunosuppresive therapy
SHARMA et al. Lancet Hematology 2021	2020	184	47	17 (range 1–243)	41,8% / NA / 15%	68% at 30 days	32% at 30 days (93% related to COVID-19)	Multivariate analysis (mortality) *50 years or older (HR: 2.57; 95%CI: 1.18 – 5.63) *Male sex (HR: 2.94; 95%CI: 1.25 – 6.90) *Development of COVID-19 within 12 months of transplant (HR 2.59; 95%CI: 1.27 – 5.26)
SCHAFFRATH et al. Transplant Cell Ther 2022	02/2020 to 07/2021	130	59	25,9 (range 0,6 - 267,6)	NA / 19,2% / 19,2%	83,8% at 100 days	7,7% at 30 days 16,2% at the end of follow-up (52% in ICUs)	Multivariate analysis (mortality): *Age >60yr (OR 5,39; 95%CI: 1.46-19,92) *Active disease (OR 4,46; 95%CI 1-19,84) *Ciclosporine (OR 8,55; 95%CI 2,24-32,63) *Time from allo-HSCT to SARS-CoV-2 detection < 1 year (OR 5,6; 95%CI 1,63-19,21) Multivariate analysis (ICU admission): *MDS (OR 4,98; 95%CI 1,22-20,35) *Ongoing immunosuppresive therapy (3,92; 95%CI 1,33-11,57) *Pandemic phase 0/1 vs 2/3 (OR 5.24; 95%CI 1.35-20.34
XHAARD et al. British Journal of Haematology 2021	2020	56	52.6	15,6 (0,4-228)	51,8% / 24,1% / NA	75% at 30 days	25% at 30 days	Multivariate analysis (severity): *Probable pneumonia *Symptoms other than respiratory *Ongoing immunosuppresive therapy Univariate analysis (mortality): *Older age at allo-HSCT *Shorter time from allo-HSCT to COVID-19 diagnosis *Probable pneumonia *Co-infection during the course of COVID-19 *Lower platelet count

°The ISI group is based on readily available clinical and laboratory data such as age, neutropenia, lymphocytopenia, myeloablative conditioning regimen use, presence of GVHD, corticosteroid use, recent HSCT, and lack of stem cell engraftment.

Hosp, hospitalisation; ICUs, intensive care unit hospitalization; MV, mechanical ventilation; MDS, myelodysplastic syndrome.Orange part: studies including (but not restricted to) allo-HSCT recipients. Blue part: studies restricted to allo-HSCT recipients.

Older age, altered WHO performance status, higher immunodeficiency scoring index (ISI) or ICU admission were predictive factors for mortality in the pooled population in the multivariate analysis. Only advanced age and ICU admission remained significant in the allo-HSCT group.

A high ISI group rate (based on advanced age, neutropenia, lymphocytopenia, use of myeloablative conditioning regimen, presence of graft-versus-host disease (GVHD), corticosteroid intake, recent HSCT, and lack of stem cell engraftment) representing a highly immunodeficient status was associated with a higher risk of ICU admission in multivariate analysis for the pooled population. In the allo-HSCT recipients’ group, only ongoing immunosuppressive therapy had a significant impact. Although immunosuppressive therapy did not have a significant effect on mortality risk in this study, it was found to be a significant risk factor of ICU hospitalization, and thus may have an indirect effect on mortality. An increased risk of severe disease was also found in other studies (4, 6) and it was associated with a higher risk of mortality in one of them (6).

In another large study from the Center for International Blood and Marrow Transplant Research (CIBMTR) database by Sharma et al. (7), involving 184 allo-HSCT recipients and 134 autologous HSCT infected by SARS-CoV-2, a specific focus on allo-HSCT recipients was performed. In this high-risk population, with a 32% mortality at 30 days, older age, male sex and SARS-CoV-2 infection within 12 months of transplant were associated with a higher mortality in multivariate analysis. Although shorter time since HSCT and older age may act as confounding factors in the assessment of mortality, due to a higher risk of non-COVID-19 transplant-related mortality, they are also associated with a more vulnerable immune system, which may explain more severe infections with various pathogens including viruses. Both studies reported an increased risk of severe disease due to immunosuppressive factors in a vulnerable population, which is consistent with the findings of other studies, as summarized in **Table 1**.

The poor prognosis reported in the first wave of winter 2019, seems to improve over time as reported in the ECIL recommendations (8) where the initial death rate of 40.7% in the first wave dropped significantly to 24.7% in the second wave of winter 2020 for patients with hematological malignancies, based on the results in the study by Pagano et al. (15). These improved results could be the reward for a better understanding of COVID-19, more effective medical management, especially in intensive care units, combined with vaccine development.

Since the breakthrough of SARS-CoV-2 more than 2 years ago, several new variants have also emerged. Currently, the Omicron variant represents the majority of COVID-19 cases. Characterized by a partial immune escape to vaccines and associated with a more important diffusion, the symptomatology seems to be less aggressive than its predecessors. Indeed, numerous studies (16–25) reported lower rates of hospitalization and death with Omicron compared to Delta or Alpha variants (HR for hospitalization: 0.25-0.41, HR for death: 0.14-0.31). Despite a less aggressive virus, Omicron is still a threat with 6-7% of death among hospitalized patients in the general population (17, 24). One study (26) reports a higher case fatality rate in patients with cancer (21% with hematological diseases) estimated at 4,9% in this study, with an OR at 2,57 (1,35-4,56) when compared with a healthy control group. For this reason, recent improvements in clinical care, COVID-19 remains a concern impacting on the management of allo-HSCT recipients.

## 3 Impact of COVID-19 on the organization of HSCT

### 3.1 A new organization of HSCT

SARS-CoV-2 pandemic had a worldwide impact, with severe consequences on hospital organization, which required multiple adaptations in daily medical practices as reported in the following studies (27–30). First, a reduction in the number of allo-HSCT was reported during this crisis in multiple countries (28), as shown in the EBMT report (29), with a decrease of 5,1% in 2020 versus 2019. These variations are in accordance with the EBMT guidelines (31) which recommended to delay, if possible, transplantations for chronic non-malignant disease. In fact, in the EBMT report (29), as well as in other studies (27, 28), the decrease impacts essentially non-malignant diseases (sickle cell disease by −30.9%, thalassemia by −19.6%) (29) and respects hematological malignancies with only a small decrease (acute myeloid leukemia decreased by −2.1%, myelodysplastic syndromes decreased by −4.3%, myeloproliferative syndromes decreased by −1.2%, acute lymphoblastic leukemia decreased by −0.7%). In all these studies, lymphoid disorders were also less transplanted, but this decrease may be due to new therapeutics, with the emergence of chimeric antigen receptor (CAR)-T cell therapies, rather than induced by COVID-19 pandemic. Moreover, there was no difference in age, sex or level of comorbidity index between patients transplanted during the COVID-19 era and those transplanted before (28), suggesting that COVID-19 did not result in the use of more stringent eligibility criteria for allogeneic transplantation.

COVID-19 has also impacted the choice of donors. In fact, during the crisis, international bone marrow donor centers faced an international traffic perturbation which may have contributed to an increase in the use domestic donors [37% vs 22% in the Deutsche Knochenmarkspenderdatei (DKMS) report (30)], easier to manage. Despite the fact that there was no difference in the United States study (28), the two European studies (27, 29) reported a higher number of haplo-identical transplants (an increase from 6.2% to 12%), with similar results in Chinese studies (32, 33). Although a family donor may be easier to manage, with more certainty about the success of stem cell collection, this increase may also be explained by better knowledge of haploidentical transplantation and better prevention of GVHD thanks to post-transplant cyclophosphamide (PTCy).

A huge decrease in the use of bone marrow (BM) grafts, as opposed to peripheral blood stem cells (PBSC), has also been observed since the breakthrough of COVID-19, with a decrease of 37% in the EBMT report. This preferential choice of PBSC grafts can be explained by closure of operating rooms during the pandemic, making the BM collection more complex. Moreover, EBMT guidelines and American Society for Transplantation and Cellular Therapy (ASTCT) guidelines published during the crisis recommended cryopreservation, which quickly became the standard. The technical difficulty to freeze and thaw allogenic bone marrow cells as compared to mobilized PBSC could also explained the increase of PBSC use. Cryopreservation was introduced to prevent two major risks. The first one is collection failure caused by a SARS-CoV-2 infected symptomatic donor or a travel incident, and thus the potential lack of available graft for a patient who already started receiving the pre-transplant conditioning regimen. The second risk is the putative transmission of SARS-CoV-2 through the graft, which may be prevented by a delaying transplantation in order to watch the evolution of donor’s condition during the few days following collection.

### 3.2 SARS-CoV-2 transmission risk through blood or stem cell donation

The theoretical risk of transmission through blood products comes from the report of a viremia detected in 15% of patients in a Chinese study (34). The demonstration of such a viremia has led to a questioning of the safety of blood transfusion. Several studies have already reported different frequencies of viremia detection, with a risk of 1,03.10^-4^ in a random screening of blood donations (35), 3,4% in a systematic testing of blood donations from symptomatic or nasopharyngeal PCR-positive patients (36), and 1,1% in blood products from donors testing positive for SARS-CoV-2 or developing COVID-19-like symptoms within 15 days after donation (all of them with high Ct values, above 37 cycles) (37). None of these three studies succeeded to find a cytopathogenic effect in blood culture (35–37). Moreover, several studies (35–44) have reported the outcome of patients transfused with blood donations from SARS-CoV-2 positive donors (at the time of blood collection or shortly after), and none of them found evidence of a blood transmission, including among immunosuppressed patients. These studies are not in favor of a possible blood transmission with a non-symptomatic infected donor, because of a rare frequency of viremia with low viral loads and without biological cytopathogenic effect, suggesting a good prevention of the SARS-CoV-2 transmission by the simple eviction of the symptomatic donors from blood collections without systematic screening.

Few studies have reported cases of PBSC infusion from positive donors at time of collection (without cryopreservation) or shortly after donation, despite negative screening prior to the start of bone marrow stimulation. Despite the donor’s positivity, no transmission was proven in recipients, with all PCR screening tests being negative in the early days following HSC infusion (43, 45, 46). Importantly, all donors were asymptomatic at the time of cell collection, which could be associated with a lack of viremia or a low viral load. One case concerned a bone marrow graft in which no virus was detected (46). This data is reassuring about the risk for the recipient but still poorly documented. Moreover, little is known about the COVID-19 complications that may be triggered by the use of granulocyte colony-stimulating factors (G-CSF) in a SARS-CoV-2-infected donor, which is consistent with the need of a negative SARS-CoV-2 PCR screening test for the donor before starting hematopoietic stem cell (HSC) mobilization, as required by some, but not all, donor registries and as recommended by EBMT guidelines.

### 3.3 Impact and interest of stem cell cryopreservation

As mentioned above, during the first wave of COVID-19 pandemic in March 2020, cryopreservation of harvested HSCs became a standard of care, although associated with a theoretical negative impact on graft quality it can lead to. This issue was already addressed in multiple studies before the COVID-19 pandemic, showing no significant difference in overall survival (OS) nor in progression-free survival (PFS) between fresh and cryopreserved grafts (47–51), including cases with GVHD prevention by PTCy (52). Similar results were reported in studies conducted during the COVID-19 pandemic (53, 54). Despite non-significant results, a part of these studies reported a trend toward worse clinical outcome when using cryopreserved products. The lack of statistical significance could be related to small patient populations in each study or to selection and confusion biases related to their retrospective design. Moreover, one retrospective study limited to severe aplastic anemia patients (55) found after adjusting for sex, recipient CMV serostatus, performance score, comorbidity index, and ABO blood group match, a significant difference in adjusted overall survival at one year: 73% for the cryopreserved group vs 91% with fresh grafts. Another large retrospective study among 1883 patients (56) found an increase in mortality and relapse, resulting in decreased overall survival at 2 years (46% vs 57%, in the cryopreserved and fresh unrelated PBSC grafts respectively). Such differences were not observed for matched related PBSC grafts and bone marrow grafts. This trend could be explained by more prolonged cytopenic-related complications, with a delay of a few days for neutrophil (ranging from 0 to 7 days) and platelet (ranging from 3 to 9 days) engraftment with cryopreserved PBSC (53–56) or a possible increased risk of graft failure, which has been suggested to be nearly twice as high in some studies (53–55) (8,9% vs 3,4% in Maurer’s study), although this difference is not significant. These complications could be related to an impact of cryopreservation on cell viability, which is generally greater than 70% for CD34+ cells (51, 57–60), but appears to be worse for other lineages such as megakaryocyte colony-forming units (approximately 40% vs. ⩾ 70% for other units in the study by Kim et al. (51) or have an impact on the phenotypic expression of cells, especially regulatory T cells (Tregs) leading to a possible excess risk of GVHD with a cryopreserved graft, as reported in some studies (49, 50, 53, 56). All these studies share a common bias related to a control group chosen outside of the COVID-19 pandemic. Although these results are based on retrospective studies, they represent a first warning regarding the use of cryopreservation in allo-HSCT, and prospective studies are needed, like the Société Francophone de Greffe de Moelle et de Thérapie Cellulaire (SFGM-TC)/Agence de Biomédecine (ABM) study which will start during 2022.

To summarize, cryopreservation is a useful tool during the COVID-19 pandemic to prevent the risk of viral transmission, but also to prevent graft collection failure for a patient who started conditioning regimen. It quickly became a standard thanks to the greater manageability it offers. Currently, with a better knowledge of COVID-19, the rise of less aggressive variants, and a possible better outcome after receiving a fresh versus cryopreserved graft, it seems reasonable to prefer a fresh graft whenever possible, notably for family donors or for bone marrow transplantation.

## 4 Vaccination, an important turn in COVID-19 history

SARS-CoV-2 is an RNA virus, composed of four main parts: a nucleocapsid, an envelope, a membrane and a spike protein (S protein). Carrying a receptor-binding domain (RBD), the S protein is responsible for the fusion of the virus with the infected cell membrane by binding to the angiotensin-converting enzyme 2 (ACE2) receptors in human cells.

Many types of vaccines have appeared since the breakthrough of COVID-19, based on the spike protein. Currently, five vaccines are approved in the European Union. The first type is mRNA-based vaccines, including Moderna/mRNA-1273 and Pfizer/BNT162b2. The second is a non-replicating competent adenovirus vector vaccine, including AstraZeneca/ChAdOx1-S and Johnson & Johnson/Jansen/Ad26.COV2-S. The last one is the recombinant nanoparticle protein-based vaccine Novavax/NVX-CoV2373. Although all have proven efficacity, mRNA vaccines have been the most widely used in allo-HSCT recipients, making them the best known in terms of safety in this population. Therefore, the EBMT has recommended the use of mRNA vaccines if possible (61). BNT162b2, in which the mRNA encodes the RBD of S protein, was the first to show a clinical efficacy against SARS-CoV-2 infection in a phase 3 study (62) with a protection rate of 95%. Similar results were obtained with mRNA-1273 [94,1% of vaccine efficacy (63)] which is an mRNA-based vaccine encoding a stabilized pre-fusion form of SARS-CoV-2 S protein.

All current vaccines are based on the spike protein, but over time, various SARS-CoV-2 mutations have occurred, resulting in the emergence of multiple variants, the most frequent being nowadays the B.1.1.529 (Omicron) variant. Multiple mutations have been found in the spike protein, primarily in the RBD domain (64), leading to a more contagious virus with immune escape (65, 66). This immune escape was clearly observed in reinfected cases and vaccinated cases but appeared to be less severe in vaccinated patients (26, 67, 68), which may be related to persistent partial protection after vaccination. In the following sections, we will focus on the vaccine-induced humoral and T cell responses and the interest of donor vaccination

### 4.1 Humoral response

#### 4.1.1 In the general population

The humoral response has been studied since the beginning of vaccine development and is often compared with post-infection immunity. SARS-CoV-2 infection results in the development of neutralizing antibodies (Nab) targeting several antiviral proteins, including the S protein, the RBD domain of the spike protein, and the nucleocapsid core (N) (which can be used to differentiate between a resolving infection and vaccination). Although initially protected in the first few months after infection (69, 70), patients progressively lose their immunity (both humoral and cellular) over time, especially beyond one year after infection (71, 72). However, some of this immunity can be restored or maintained by vaccination, as suggested by the increase in antibody levels (69), highlighting the value of vaccination, including in previously infected patients. Moreover, Dimeglio et al. (73) reported, in their study, a possible advantage of previous infection in vaccinated patients with a more persistent immune protection in post-infection patients (Ac>141BAU/ml for 603 days in previously infected versus 309 days in non-infected vaccinated patients after the last vaccine injection) (73). These differences may be related to a better cellular response in infected patients, a better antibody affinity, or a greater immune stimulation by the virus itself which could lead to a better memory-cell persistence.

#### 4.1.2 In HSCT patients

Currently, vaccination remains the first line of prevention against SARS-CoV-2 infection despite the appearance of new variants. For these reasons, it is important to identify factors predictive of humoral response, particularly in vulnerable populations like allo-HSCT patients [**Table 2** (74–90)]. In these studies, the conditioning regimen is most often of reduced intensity rather than myeloablative, and donors are more likely to be unrelated. Approximately one-third of patients are on immunosuppressive therapy at the time of or within 3 months before vaccination, nearly 50% have a history of GVHD, and approximately 20-25% have chronic GVHD. The median time between transplantation and vaccination varies between studies, ranging from 14 months to 98 months, with no patient having received a transplant within 3 months, with rare exceptions. Most of them were vaccinated with BNT162b2 or mRNA-1273, mRNA vaccines.

**Table 2:** Predictive factors of reponse to SARS-CoV-2 vaccines in Allo-HSCT patients; adapted from Chaekal et al, Transplantation and Cellular Therapy 2022.

Author	Population	Age	Donor type				Conditioning regimen		IS (at time of vaccination or during the previous 3 months)	GVHD	Time from transplantant to vaccination (months)	Vaccine type and number of doses	Response	Risk factors for vaccine response	
			MRD	MURD/MMURD	Haploidentical	UCB	MAC	RIC						Univariate analysis	Multivariate analysis
MAILLARD, Blood letter, 2022	n: 687	59 (IQR 46-66)	194 (29.6%)	333 (50.8%)	129 (19.7%)	0	196 (31.3%)	430 (68.7%)	203 (30.0%)	239 (38.4%) (history of GVHD requiring systemic treatment)	27 (21% < 12m)	mRNA-1273 BNT162b2 2 doses	*78% Ab+ *mean level: 749 BAU/mL *61%>250BAU/mL	*Time since transplant *Ongoing antineoplastic treatments *Ongoing immunosuppressive treatments (< 3 months) *Rituximab (< 6 months) *Absolute lymphocyte count (<1G/L) *T-CD4+ cell count (< 500/mm3) *B-CD19+ cell count(100/mm3) *Gammaglobulinemia (< 6gr/L)	Model A: *Time since transplant < 1 year OR 2.73 (1.63 to 4.59) *Immunosuppressive treatments (< 3m) OR 3.41 (2.09 to 5.55) *Rituximab (< 6 months) OR 13.61 (4.1 to 45.19) *Absolute lymphocyte count < 1 G/L OR 3.09 (1.88 to 5.09) Model B: *Time since transplant < 1 year OR 4.67 (2.41 to 9.07) *Immunosuppressive treatments (< 3m) OR 2.75 (1.37 to 5.54) *B-CD19+ cell count < 100/mm3 OR 5.72 (2.75 to 11.87)
PINANA, American Journal of Haematology, 2021	n: 311	56.7 (18–80)	127 (41%)	102 (33%)	76 (24%)	6 (2%)	133 (43%)	178 (57%)	104 (33%)	82 (26%) (active cGVH at vaccination)	98 (4–646)	mRNA-1273: 261 (84%) BNT162b2: 47 (14%) AZD1222: 2 (0.6%) Ad26.COV2.: 1 (0.3%) 2 doses	*78% Ab+	*Active GvHD *Lymphopenia < 1G/L *Immunosuppressive treatments *Steroids	*Lymphopenia < 1G/L OR 0.33 ( 0.16;0.69), *Active graft versus host disease (GvHD) OR 0.51 ( 0.27;0.98) *Vaccination within the first year of transplant OR 0.3 (0.15;0.9)
BEERLAGE Transplant infectius disease, 2022	n: 182	56 (21-80)	65 (35.7%)	98 (54,5%)	16 (8.7%)	2 (1.1%)	NA	NA	77 (42.3%)	93 (51.1%) (including acute or chronic GVHD)	39 (3-408)	mRNA-1273: 96 (52.7%) BNT162b2: 86 (47.3%) 2 doses	*91,8% >50 U/mL *75,3% >250 U/mL	*Time since transplant *Absolute lymphocyte count (< 0,9G/L) *Ongoing immunosuppressive treatments (< 3 months) *Ongoing tumor treatment	NA
PABST, Vaccines,2022	n: 167	60 (19-79)	NA	NA	NA	NA	NA	NA	60 (35,9%)	NA	40 (3,5-299,5))	AZ/AZ: 15 (9.0%) AZ/Moderna: 1 (0.6%) AZ/Biontech: 11 (6.6%) Biontech/Biontech: 133 (79.6%) Moderna/Biontech : 1 (0.6%) Moderna/Moderna: 6 (3.6%) 2 doses	*81% Ab+	NA	*Short time since transplant OR 0,174 (0,033;0,926) *Ongoing⩾2 IS OR 0,08 (0,001; 0,119) *Older age OR 0,002 (0,000;0,808) *Higher B-cell lymphocytes OR 2,843 (1,052;7,683) *Time interval from vaccination OR 0,126 (0,028;0,0559) *Second vaccine is mRNA OR 6,185 (1,019;37,534)
CHAEKAL, Transplantation and cellular therapy, 2022	n: 145 Allograft and Alemtuzumab (MRD/MUD) or ATG (haplo-cord)	61 (24-80)	47 (32%)	53 (37%)	45 (31%)		13 (9%)	48 (91%)		22 (15%) (cGVHD)	31 (3-111)	BNT162b2: (61%), 2 doses mRNA-1273: (33%), 2 doses J&J (5,5%) 1 dose	*85% Ab+ *68% >100 BAU/mL	*Age *Time since transplantation < 12m *Chronic GVHD *Rituximab history before transplantation *Absolute lymphocyte count (< 1G/L) *Antitumoral treatments	*Time since transplant > 12m OR 3,85 (1,43;10) *No Chronic GVHD OR 5,6 (2,13;14,2) *Absolute lymphocyte count (> 1G/L) OR 4,01 (1,63;9,92)
TAMARI, Blood Cancer Discov, 2021	n: 149 Allograft n: 61 AutoHCST n: 7 CarT-cells	66.4 (25.8–84.1)	NA	NA	NA	NA	NA	NA	Allograft only: 46 (30,8%)	NA	Allograft only: 36,5 (IQR: 18,8-67,1) <12m: 19 (12,7%)	BNT162b2: 152 (70%) mRNA-127: 65 (30%)	Allograft only: 89% Ab+	*Gammaglobulinemia < 5gr/L *CD19+cells < 50/µL *CD4+cells < 200/µL *Steroids	*CD4+cell count ≥ 200µL, OR: 1.08 (0.96;1.22) *CD19+cell count ≥ 50/µL, OR: 1.53 (1.32;1.76) Gammaglobulinemia ≥ 5gr/L, OR: 1.15 (1.02;1.3)
JULLIEN, Transplantation and cellular therapy, 2022	n: 117	57.1 (44.2-65.9)	Matched related and unrelated: 79 (67.5%)		36 (30.8%)	NA	23 (19.7%)	87 (74.4%)	32 (27.4%)	62 (53.0%) (GVHD history)	21,5 (IQR: 12,2-45)	BNT162b2: 100% 2 doses	*83% Ab +	*Time since transplant *Haploidentical graft *Ongoing immunosuppressive treatments or tumor treatments *Absolute lymphopenia *T-CD4 lymphopenia *B-cell lymphopenia	*B-cell aplasia, OR 0,01 (0,00 ; 0,1)
LE BOURGEOIS, Jama 2021	n: 117	57 (20-75)	30 (25,5%)	51 (44%)	36 (31%)	0	NA	NA	26 (22,2%)	62 (53%) (history of GVHD)	21,5 (3-203,8)	BNT162b2: 100% 2 doses	*83% Ab+ *62% >Higher level	*Time since transplant < 12m *Haploidentical graft *Ongoing immunosuppressive treatments or tumor treatments *Absolute lymphocyte count (< 1G/L)	NA
CHEVALIER, eJHaem, 2021	n: 112	57 (20–75)	26 (23,2%)	51 (45,5%)	35 (31,2%)	0	23 (20,5%)	83 (74%)	25 (22,3%)	57 (51%) (history of GVHD)	21,8 (3–203,8)	BNT162b2: 100% 1 dose	*55,35 Ab+	*Time since transplant *Absolute lymphocyte count (< 1G/L) *Ongoing immunosuppressive treatments or tumor treatment	NA
HUANG, Transplantation and cellular therapy, 2022	n: 110	57 (46- 65)	32 (29.1%)	57 (51.8%)	21 (19.1%)	0	36 (32.7%)	74 (67.3%)	51 (46,4%)	26 (23.6%) (cGVHD moderate to severe)	20 (3-420) 39,1% between 3-12m	BNT162b2: 94 (85.5%) mRNA-1273: 16 (14,5%) 2 doses	*62% with NT50 >250 (neutralization titers)	*Age > 65 years *Immunosuppression *Relapsed disease *Time since transplant < 12m *CGVH*Pregraft covid infection	*Age > 65 years OR: -0.59 (-1.11 ; -0.07) *Immunosuppression OR: -0.44 (-0.84 ; -0.04) *Relapsed disease OR: -0.55 (-0.98 ; -0.12) *Time since transplant < 12m OR: -0.66 (-1.06 ; -0.25)
YESHURUN, Blood, 2021	n: 106	65 (23-80)	41 (39%)	61 (58%)	4 (3%)		69 (65%)	37 (35%)	52 (49%)	75 (71%) (history of GVHD)	42 (4-439)	BNT162b2: 100% 2 doses	*85,8% Ab+	*GVHD moderate/severe *Time since transplant (< 4,5y) *Ongoing immunosuppresive treatments	*Time since transplant (< 4,5y) OR 10,8 (1,2 ; 93,2) Subgroup analysis (time since transplant (<4,5y)) *Immunosuppresive treatment OR 10 (1,1-93)
REDJOUL, The lancet, 2021	n: 88	60 (26-77)	26 (29,5%)	46 (52,3%)	16 (18,2%)	0	NA	NA	38 (43,2%)	44 (50%) (history of GVHD requiring systemic treatment )	23 (3-213)	BNT162b2: 100% 2 doses	*78% Ab+ *59% >4160AU/mL	*Time since transplant < 12m *Absolute lymphocyte count (< 1G/L) *Ongoing immunosuppressive treatments (< 3 months)	*Absolute lymphocyte count (> 1G/L): OR 6,4 (1,2-34) *Ongoing immunosuppressive treatments (< 3 months) OR 0.07 (0.02-0.25)
MORSINK, Blood Cancer Journal, 2022	n: 70 with Endoxan post-HSCT	62 (24–76)	10 (14%)	51 (73%)	9 (13%)	0	49 (70%)	21 (30)	12,4 (20%)	NA	27.8 (0.6–49.5)	mRNA-1273: 54 (77%) BNT162b2: 8 (11%) Combination mRNA-1273/BNT162b2: 6 (9%) Other: 2 (3%) 2 doses	*87% with titer > 300 AU/ml	*Ruxolitinib *Ibrutinib	NA
TSUSHIMA International Journal of Haematology, 2022	n: 65	55 (23-80)	16 (24,6%)	31 (47,7%)	15 (23%)	0	39 (60%)	26 (40%)	28 (43.1)	22 (33.8%) (aGVHD or cGVHD)	92 (4-255)	mRNA-1273: 2 (3,1%) BNT162b2: 62 (95.4%) 2 doses	*87,7% Ab+ *76,9% >260 U/mL	*Time since transplant *Active GVHD *Ongoing immunosuppressive treatments *Absolute lymphocyte count (< 1G/L) *T-CD4+ cell count (< 500/mm3) *B-CD19+ cell count(< 100/mm3) *Gammaglobulinemia (< 6gr/L)	NA
MAMEZ, Bone Marrow Transplant, 2021	n: 63	54 (18-78)	NA	NA	NA	NA	NA	NA	NA	NA	14 (3-150)	mRNA-1273: 46 (73%) BNT162b2: 17 (27%) 2 doses	*78% Ab+ *Ab median level: 815 U/mL	*Age > 60ans *Time since transplant *ATG *Absolute lymphocyte count (< 1G/L)	*Age > 60ans: OR -1,72 (-3,43;-0,28) *Time since transplant (> 6m) OR 1,97 (0,46; 3,7) *ATG: OR -2,66 (-5,8; -0,65)
CANTI, Journal of hematology and oncology, 2021	n: 40	60 (26-76)	8 (20%)	27 (67,5%)	5 (12,5%)	0	NA	NA	14 ( 35%)	11 (27,5%) (chronic GVHD ⩾ moderate)	31 (6-57)	BNT162b2: 100% 1 dose	*86% Ab+	*GVHD moderate/severe *Time since transplant *Rituximab < 1an *Age	*GVHD moderate/severe OR-1,18 *Long time since transplant OR 5,72 *Rituximab < 1 year OR -0,97 *Age OR -0,027
WATANABE, Vaccines, 2022	n: 25	55 (23-71)	NA	NA	NA	NA	9 (36%)	16 (64%)	7 (28%)	NA	53 (5-137)	BNT162b2: 100% 2 doses	*76% Ab+	*Gammaglobulinemia (< 6gr/L) *Steroids *Ongoing immunosuppressive treatments	NA

GVHD, graft vs host disease including chronic, GVHD (cGVHD) and acute GVHD (aGVHD). MRD, matched related donor; MURD/MMURD, matched / mismatched unrelated donor; UCB, umbilical cord blood; MAC, myeloablative conditioning; RIC, reduced intensity conditioning; ATG, anti thymocyte globulin; Ab+, detectable anti-SARS-CoV-2 antibodies. Model A: adjusted with available lymphocyte count; Model B: adjusted with gammaglobulinemia and CD4+ T cell and CD19+ B cell counts.

First, despite a lower response rate than a control group of healthy workers (78, 84, 85, 88, 90, 91), more than 75% of patients have a positive serology after two doses, and about 60% have antibody levels above the effective protection level defined at the time of the studies.

Second, in a study of T cell depletion conditioning with anti-thymocyte globulin (ATG) or alemtuzumab (83), it is also reported that 85% of patients achieve seroconversion and 68% achieve a good response rate (antibody level >100BAU/mL). However, ATG was found to be a negative predictor of response in multivariate analysis in a single study with 63 patients (79), where it is important to note the short delay between transplantation and vaccination in this study (median 14 months), which may imply an impact of ATG on the response to vaccination in the early post-transplant period, especially since this result was not specifically found in the other studies with a much longer median delay. However, these same studies have reported lymphopenia and immunosuppressive drugs as factors of poor response, both of which indirectly included ATG or its lymphopenic effect.

The frequently identified detrimental factors are related to an impact on the immune system or its reconstitution after transplant. In fact, a time of less than 1 year after allogeneic stem cell transplant, the use of immunosuppressive therapy (including steroids) or the use of rituximab, are described as impact factors. Similarly, GVHD appears to be an inconsistent factor in multivariate analyses. These results may be related to a greater impact of GVHD treatments with the use of immunosuppressive therapies combined with steroids than GVHD itself.

Multiple studies investigated lymphopenia, which may be a surrogate marker of immune system capacity, and consider it a major pejorative factor in humoral response, often at the threshold of <1 Giga/L. If lymphocytes are studied separately as B cells, T cells, and gamma-globulinemia representing plasma cell activity, many studies report an independent impact of the B cell population only (74, 80, 81). One of these studies (80) goes as far as to report B cell aplasia as the strongest predictor of a poor humoral response, with a seroconversion in 9.1% in B cell aplasia group versus 95.9% in the control group.

Taken together, these results are consistent with EBMT recommendations to start vaccination of allo-HSCT recipients after 3 months post-transplant if they have not received anti-CD20 treatment during the last 6 months and do not suffer uncontrolled GVHD.

To improve the humoral response in allo-HSCT recipients, a third early dose of vaccination has been proposed. **Table 3** summarizes the results of the main studies of a third vaccine dose in this population (74, 92–97). Good responses are observed for most patients (48-85%), with a seroconversion around 40-50% in patients who had not responded after 2 doses. Analysis of predictive factors was performed in only some of these studies, with small numbers of patients, but the results are similar to those of the studies mentioned in **Table 2**, as they also report a negative impact of low B cell counts or the use of rituximab or, at a least degree, other immunosuppressive therapies. In the study by Canti et al. (95), the mean value of neutralizing antibodies (Nab) increased at d28 after the third dose for wild type (from 52,5 to 566,8) and Delta viruses (from 28,8 to 200,4). 60,5% of patients also have Nab against Omicron, at a median of 80,5, which is lower than for other viruses. Overall, these studies support early administration of a third booster vaccine dose to poor responders after two doses.

**Table 3:** Impact of third dose in allo-HSCT patients.

Author	Type	Population NR/LR/GR	Median time between V2-V3 (days)	Response evaluation (around 30d aftervaccination)	Specific response	Risk factors predictive of vaccine response	
						Univariate analysis	Multivariate analysis
MAILLARD, Blood letter, 2022	Early third dose	n:181 NR : 70(38,7%) / LR : 46(25,4%) / GR : 65 (35,9%)	54d	NR / LR / GR: 41(22,6%) / 36 (19,9%) / 104 (57,5%)	NR=> 41%LR LR=> 85%GR		
REDJOUL, The lancet, 2021	Early third dose	n:42 100% Ab- (Ab <4160 AU/mL)	51d	22 (52%) Ab- / 20 (48%) Ab+	Ab- => 48% Ab+	*B-cell count >0,25G/L *IGG antisarscov before V3 >1000 AU/mL	*B-cell count >0,25G/L OR 7,1 (1,5;34,1)
BILAL ABID Cancer cell, 2021	Early third dose	n:26 100% NR		15 (58%) ⩾LR	58% seroconvert	No factor found	
CHEVALIER, Hematological Oncology, 2022	Regular booster	n:121 No data before V3		NR / LR / GR: 14 (11%) / 7 (6%) / 103 (83%)		*High Lymphocyte count *High B-cell count *High T-cell count *Time between transplant and first vaccine >12m *No IS *No chemotherapy	
LE BOURGEOIS, British journal of haematology, 2021	Regular booster	n:80 No data before V3		NR: 9 (11%) Increase: 19 (24%) Decrease/stable: 4 (5%) Highest level: 48 (60%)	2 seroconvert 17 ( 35%) reach the highest level		
KIMURA, Transplantation and cellular therapy, 2022	Regular booster	n: 64 No data before V3		NR / SR / R: 10,9% / 18,8% / 70,3%		*Chronic kidney disease *Haploidentical donor *Low lymphocyte count	*Haploidentical donor OR 7,67 (1,86-31,60)
CANTI, Cancer cells, 2022	Regular booster	n:38 No data before V3	132d	*Median of 152 BAU/mL to 2955 BAU/mL *Patients with Nab WT/Delta/Omicron: Before: 40% / 13% / Unknown After: 87% / 82% / 60,5%	4/5 seroconversion	*Moderate/severe chronic GVHD *Rituximab administration within the year before first vaccine dose	

NR, no reponse with negative serology. LR, low response with an antibody level <250 BAU/mL but detectable. GR, good response with an antibody level ⩾250 BAU/mL.

SR: suboptimal responder, anti-RBD titer <100UI/ml. R: responder anti-RBD titer >100UI/ml. Ab:Antibody titer. Ab+: ⩾4160 AU/mL. Nab: neutralizing antibodies, Nab titers that reduced the number of infected wells by 50% (NT50) were used as a proxy for the NAb concentration in serum. IS: immunosuppresive treatment. V2: 2nd injection. V3: 3rd injection.

Finally, two studies investigated the persistence of humoral response at 6 months after the last vaccine injection (mostly 3-doses schema). In the first study, Chevalier et al. (96) followed 141 transplanted patients who received 3 doses of the BNT162b2 vaccine (median time between the second and third dose was 44 days (20-205d)). In this cohort, 20% of the patients had a delay between transplantation and the first vaccine dose of less than 12 months. Six months after the third dose, a significant decrease in antibody levels was observed in one-third of patients. Sixty-nine percent (54/78) of patients who had a good response at initial evaluation still had a good response at 6 months. Three patients became infected by SARS-CoV-2 during the 6-month follow-up, although two of them had a good response to vaccination. They developed mild/moderate symptoms while the last one, with low humoral response, died of infection.

Another study by Leclerc et al. (98) reported the same decrease in antibody levels at 6 months with a nearly three-fold reduction in specific IgG titers. Despite this decrease, 72% of patients maintained a protective antibody level at 6 months. There was a correlation between the peak antibody value one month after the last vaccine dose and the residual antibody level at 6 months, with a threshold of more than 10,000 AU/mL at peak ensuring a protective level of more than 4160 AU/mL at 6 months. In univariate analysis, factors associated with low antibody levels at 6 months (<1000 AU/mL) were rituximab infusions given within 6 months before vaccination, systemic immunosuppressive drugs given within 3 months before vaccination, as well as low lymphocyte count <1 Giga/L, and B cell count <0.25 Giga/L at time of vaccination. Four SARS-CoV-2 infections were also reported during follow-up, two of these occurring among patients who had a good response after 2 doses. Only one patient, with a poor response at the time of infection, died. The results of these two studies are in favor of a waning of immune protection, as illustrated by the progressive decrease in antibody titers over time.

To conclude, with the rise of new variants and the need for a higher level of antibodies than for wild-type SARS-CoV-2, the issue of the need for regular vaccine boosters in all vulnerable patients, regardless of their response, should be discussed within a time frame that remains to be defined (every 6 months?).

### 4.2 Cellular response

Although humoral response was soon found to efficiently prevent SARS-CoV-2 infection, few studies also reported an important action of CD4+ and CD8+ T cells in the resolution of COVID-19 or even on its mortality (99–101). Indeed, early studies of vaccine efficacy reported both humoral and cellular responses in healthy patients. Although most studies in allo-HSCT recipients have focused on humoral responses, some have also investigated the cellular response. As described with humoral response, the rate of patients with a cellular response and the level of that response are lower than in a healthy control group but increased with the used of repeated doses of vaccine (102–105). Indeed, the use of two doses achieves a level of T cell response (based on interferon-γ or TNF-α production by SARS-CoV-2 antigen-specific CD4 or CD8 T cells) in approximately 80% of patients, as compared with 30% after a single dose, associated with a larger pool of persistent memory T cells (103, 104, 106). Inferior results in allo-HSCT recipients as compared with a healthy control group could be explained, at least in part, by a more immature immune system with more frequent lymphopenia and the use of immunosuppressants or steroids, the same predictive factors as those reported in humoral response studies.

Jarisch et al. (107), addressed the issue of the correlation between humoral response and T cell response. There was a lower rate in T cell response in the humoral non-responder group after 2 doses, but 41% of these patients succeeded to produce a T cell response. In addition, in a B cell aplasia group, including 2 allo-HSCT recipients and 6 CAR-T cells patients, a cellular response was detected in most patients despite the absence of a humoral response after a third vaccine dose, suggesting a possible protective cellular response in some non-humoral responders after vaccination.

In addition, two other studies have shown that the cellular response may be predictive of humoral responses. In the first one (102), T cell response, as measured by the level of INF-γ production after stimulation with S1 peptide one month after a second dose, was predictive of a seropositivity level greater than 100 BAU/mL at 6 months (88% vs. 46% in non-responders). In the second study, Bergamaschi et al. (108), found a correlation between cytokinine profiles released by T cells and humoral response. In this study, the ability to upregulate IFN-γ and IP-10/CXCL10 during primary vaccination and upregulate IFN-γ, IL-15, IL-7 and IL-10 after the second dose were predictive of a good humoral response. These correlations highlight the importance of T cell and B cell interaction for optimal vaccine response, which can be easily deregulated in allo-HSCT patients who present with an immature immune system, in relation with lymphopenia or immunosuppressive treatments.

The T cell response is also important in the setting of the global spread of Omicron, whose immune escape from anti-S antibodies requires a higher level of antibodies to prevent infection. Two studies (109, 110) reported that ancestral SARS-CoV-2 specific T cells (obtained after vaccination) also recognize the Omicron variant, but with less intensity than the ancestral variant. This recognition is improved using a third booster dose leading to a better cellular response, which may help to reduce the severity of Omicron infection, although here again an immune escape mechanism may appear.

### 4.3 Safety

SARS-CoV-2 vaccines are generally well tolerated by allo-HSCT recipients, with predominantly mild to moderate symptoms (78, 78, 85, 87–91, 97, 111–113). More than 50% of patients experienced adverse events, ranging from 48% to 80% between studies, with mainly local reactions at the injection site (30% to 86% of patients) involving pain, redness or swelling. Systemic symptoms may also occur, such as asthenia (in 20-41% of patients), myalgia (15-30%), headache (15-30%), chills (7-15%). Although two studies (87, 89) reported a decrease in adverse events with the second dose, others reported a similar or higher rate after the second injection (90, 91, 111–114). Interestingly, in studies (88, 111) comparing allo-HSCT recipients and a healthy control cohort, a lower rate of adverse events was observed in the allo-HSCT population, likely due to a lower level of immune response.

Although adverse events were mostly mild to moderate, some patients experienced more severe symptoms. Two patients (1%) in the cohort reported by Pabst et al. (81) suffered from a herpes zoster at D7 and D14 of vaccination, an event that has been previously described in healthy vaccinated or COVID-19 infected patients (115, 116). Another important complication was reported in the study by Ram et al. (114), in which 10% of patients acquired cytopenias, mostly grade 1-2 and resolving within two weeks. However, 4 patients (5%) still developed grade 3-4 thrombocytopenia (3 patients) or grade 4 neutropenia (1 patient). However, there were important biases in this study. First, it was a mixed population including 17,5% CAR-T cells patients. Second, 8% of the patients were on active chemotherapy, which may have contributed to the cytopenias observed in this study. Similar results were reported by Ali et al. (91), with about 10% of transient cytopenias, mostly grade 1-2, but here again, 5 of these patients (5%) were on chemotherapy.

An important warning point is the potential impact of vaccination on the risk of GVHD triggering or exacerbation. Recent studies had reported a frequency of post-vaccination GVHD ranging from 3.5% to 9% (81, 82, 91, 97, 114). Most patients in these studies developed an exacerbation of GVHD within 3 months of vaccination, the majority within a month following the vaccine injection. GVHD is often described with gastrointestinal, cutaneous, or oral localization and having required systemic steroid treatment and resumption or increase of immunosuppressive medications. The incidence of GVHD flare-up was higher in patients with a history of chronic GVHD, with a frequency of approximately 10-15% (81, 82, 114). In the study by Kimura et al. (97), which evaluated the response to three doses of vaccine, no increase in GVHD risk was observed with repeated injections (9% after 1 dose, 7,6% after 2 doses, 5.4% after 3 doses). The study also reported the new onset of acute (0.7%) or chronic (3.3%) GVHD, all of which occurred within 35 days of injection and required initiation of systemic immunosuppressive therapy. Controlled data are lacking to state a direct link between injection of anti-SARS-CoV-2 vaccines and exacerbation of GVHD, but the short delay between vaccine injection and exacerbation seen in different cohorts of allo-HSCT vaccinated patients is suggestive of such a link.

Overall, these results support good vaccine safety in the allo-HSCT population, with often mild to moderate local reactions. A possible risk of vaccine-induced GVHD exacerbation is consistent with EBMT recommendations to delay vaccination of patients with active GVHD (particularly if uncontrolled).

### 4.4 Impact of donor vaccination

Donor immune protection may be carried over from the graft to the recipient. This impact on virological risk is well known in the case of CMV infection, where better protection against CMV reactivation has been shown to be associated with a survival advantage in CMV-positive recipients transplanted from CMV-positive donors as compared with CMV-negative donors. Some studies have investigated the immune protection conferred by donor vaccination in the setting of allo-HSCT. Despite the failure to transmit immunity with unconjugated pneumococcal vaccine (117, 118), a higher response rate after vaccination of allo-HSCT patients transplanted with a vaccinated donor has been found with more immunogenic vaccines, notably with conjugated vaccines such as anti-pneumococcal (119) and anti-Haemophilus influenza type b vaccines (117, 118, 120, ). Similar results have been reported with diphtheria and tetanus vaccines (120).

Leclerc et al. (121) investigated the impact of donor SARS-CoV-2 vaccination on recipient immune response to post-transplant vaccination. In this study, patients transplanted from a vaccinated donor had a higher humoral response rate after two post-transplant vaccine doses (mean of 7492 AU/mL versus 828 AU/mL). Although patient number was small (n=30), there were no significant differences in patient characteristics, in particular all patients were on immunosuppressive therapy at the time of post-transplant vaccination and there were no differences in the number of those who had received anti-CD20 therapy, or in lymphocyte counts, including B and T cells. Furthermore, in recipients vaccinated before HSCT, as well as in those who received HSCT from vaccinated donors, no one had protective antibody levels at the first three months after transplantation (i.e. just before the first post-transplant vaccine dose), suggesting that passive transmission of humoral immunity from the donor to the recipient was insufficient for effective protection. Similar results were reported by Jullien et al. (122), with a higher humoral response in the vaccinated-donor group (representing 9 of 17 patients studied). In their study, La Rosa et al. (123), reported the transfer of passive cellular and humoral immunity from the donor to the recipient. After a selection of three vaccinated donors (with two of them who probably had asymptomatic COVID-19 based on the presence of anti-N antibodies), they analyzed the transfer of immunity in three recipients, one of whom was not vaccinated prior to transplant. They found a probable humoral transfer, by the presence of SARS-CoV-2 antibodies in the recipients, as reported by the studies above, but also a T cell immune transfer. Indeed, although a possible important bias could be led by the lack of immune analysis performed on the day of transplant in recipients to eliminate a possible asymptomatic pre-existing SARS-CoV-2 infection, the presence of CD4+ and CD8+ T lymphocytes with anti-N specificity in the recipients, strongly suggests a lymphocyte transfer from donors who have already encountered the virus to recipients through the graft. Although initially with a low level at day 30, the study reported an expansion of these T-memory and effector cells as the immune reconstitution progressed. Conversely, the humoral response represented with anti-S antibody level, still low during a long period in the early post-transplant period, probably related to a slower B cell reconstitution. In patient number 3 who received an anti-SARS-CoV-2 vaccination at day 112 and day 133 after the transplant, a boosting effect on the immune T cells and on the circulating anti-S antibody level was observed. This study has the interest to suggest a transfer of B cell but also T cell immunity, which seems to expand more rapidly than the B cells during the early recipient’s cellular reconstitution, which could bring an additional protection to these fragile patients in the early post-transplant period. Moreover, the synergic effect during the recipient vaccination could lead to a better response and protection. All these studies rely on small numbers of patients, but their results encourage the adoption of a pro-vaccination strategy for donors, especially during the pre-transplant consultation for intra-family donation. The impact of the time between donor vaccination and HSC donation on recipient response has not been studied, so no additional booster dose in the donor population prior to graft collection can be recommended beyond routine vaccination.

## 5 Treatment of COVID-19 in allo-HSCT recipients

Despite numerous treatment studies published during the last 3 years, no prospective study evaluating the efficacy and tolerance of a COVID-19 treatment was specifically conducted among allo-HSCT recipients, a high risk population of patients eligible to both preventive and early curative treatments.

The first category of treatments for COVID-19 is represented by monoclonal antibodies targeting the RBD domain of the S protein. Four drugs have been approved by the European Medicines Agency (EMA), based on significant results for prevention of progression to severe disease or death in pauci-symptomatic high-risk patients: casirivimab/imdevimab (124, 125), sotrovimab (126), regdanvimab (127), tixagevimab/cilgavimab (128). Although the first three treatments are only indicated for curative treatment, tixagevimab/cilgavimab, has also been approved as prophylactic treatment (at a dose of 300mg/300mg every 6 months), based on the PREVENT study (129), reporting a 77% reduction in the risk of infection at 3 months, with a benefit confirmed in other studies (130–132), including one with an allo-HSCT population (133). The emergence of new variants has rapidly led to a loss of efficacy (134–141) (particularly with respect to BA.5, currently the most represented variant), leaving only one indication among all these treatments for tixagevimab/cilgavimab in case of contraindication to antiviral treatment, or in case of infection with an old sensitive variant, according to current French recommendations (142).

The second therapeutic class is represented by antiviral drugs, which are the most effective treatments against the current variants (141). Symptomatic patients not requiring oxygen should be treated as soon as diagnosis is confirmed (treatment should be initiated within 3 days after symptom onset) with oral nirmatrelvir/ritonavir for 5 days. Nirmatrelvir is an antiviral agent targeting the SARS-CoV-2 3-chymotrypsin–like cysteine protease enzyme (Mpro). This strategy is based on a phase 3, double-blind, placebo-controlled study (143), conducted among 2246 symptomatic, unvaccinated, nonhospitalized adult patients at high risk for progression to severe COVID-19, where treatment with nirmatrelvir/ritonavir led to a 88.9% relative risk reduction in COVID-19–related hospitalization or death from any cause versus a placebo. Despite the excellent safety profile of this drug, the clinician must remain attentive to the risk of drug interactions, in particular with calcineurin inhibitors, and to contra-indications (severe renal or hepatic impairment). Because of its good safety profile and efficacy (144), with a significant reduction of mortality in a cohort of patients with hematological malignancies (145), remdesivir, an inhibitor of the viral RNA polymerase, may also be proposed to allo-HSCT recipients, mostly in those with low-flow oxygen requirement and if treatment is initiated within 10 days after symptom onset (8).

During the inflammatory phase of the disease, i.e. when patients require oxygen and present with increased inflammatory biological markers, treatment with dexamethasone 6 mg per day for 10 days is recommended by the ECIL group (8) (based on data from the RECOVERY trial (146) showing a significant reduction in 28-day mortality).

For severe and critical cases, the management is quite similar to the general population with the same clinical severity and follows the recommendations of the ECIL group (8).

## 6 Conclusion

Because allo-HSCT recipients are particularly frail and vulnerable to infectious complications, having experienced among the highest fatality rates reported during the first wave of the pandemic, COVID-19 led to substantial changes in clinical practice among transplant centers worldwide. However, as observed in the general population and other high-risk patients, the breakthrough of mRNA vaccines dramatically modified the impact of COVID-19 in the allo-HSCT setting. Indeed, allo-HSCT recipients can be efficiently and safely vaccinated after transplant. Although immune response rates appear to be lower among these patients as compared to healthy individuals, the use of additional vaccine doses may improve seroconversion rates and restore protective immunity. The impact of pre-transplant donor vaccination has been suggested by some studies and warrants further evaluation to help and decipher the determinants of an efficient post-transplant immune protection. The constant evolution of the virus through mutations that may drive immune escape represents a challenge for adapting vaccine strategies in the near future.

## Author contributions

JB and ML wrote the manuscript. JB, SM and ML designed and reviewed the manuscript. All authors contributed to the article and approved the submitted version.
